# Dr. G. V. Black’s Conclusions Reviewed

**Published:** 1897-12

**Authors:** R. R. Andrews

**Affiliations:** Boston, Mass.


### DR. G. V. BLACK'S CONCLUSIONS REVIEWED.[1]

BY DR. R. R. ANDREWS, BOSTON, MASS.

THE invitation to take part in the discussion of the views of Dr. Black I have accepted, but not without considerable hesitation.

These are problems for the profound thinker and the conscientious student. The views have been presented to us after a series of exact experiments, so minute, in fact, that it seems almost superfluous to question them. It is well-nigh impossible to exaggerate the value of this series of papers to the members of the dental profession, and I think I speak the professional opinion throughout this nation, as well as in Europe, when I say it is unanimous in regard to the value and the magnitude of Dr. Black's contributions to the science of dentistry.

---

[1] Read before The New York Institute of Stomatology, October 5, 1897.

But the investigation, although great, cannot yet be considered complete. There is still *something wanting to make it harmonize with the experiences of our oldest and best-tried practitioners.* What thought I have given to the subject comes to me wholly from the mental impressions that are revealed not only to me, but to every thoughtful operator, as the result of many years of active practice. In this discussion, by your courtesy, I shall consider briefly the part played by the vital force through the organic matrix of the tooth. In this series of investigations on the physical character of the human teeth one gets the impression that there has hardly been given to the organic structure of the dentine and the cementum the great importance this tissue bears to the strength of the tooth and to its power to resist decay at the different periods in the life of the patient. The inorganic structure of the tooth may not have the strength in middle life that it has in youth, as has been shown by Dr. Black's experiments, but the vital power given to its organic structure by reason of robust health may be much greater, and as a result we find the organic structure fortified with this vital force, so that it will be found resisting the inroads of the destroying organisms to a very great degree. This is a phenomenon we cannot study with exact mechanical appliances. The teeth in health have a high power of resistance to decay. Who of us has not noticed the warty, discolored, and almost enamelless teeth that, strong but ugly, have resisted decay for years? And in some instances we have found decay to be completely stopped by the action of the organic structure fortified with a strong vitality. In youth, with lessened vital power, the tooth which Dr. Black's experiment shows to be stronger would, as a result, melt away. Every thoughtful operator has found that there are periods of decay, and we have all of us had experience with the results that come from overwork, fevers, or any troubles which tend to lessen this vital force. In fact, there is an abundance of testimony showing that premature or excessive intellectual work reacts upon the normal condition of the teeth, and scholastic success is often rewarded by impaired health and dental caries.

Some of us are familiar with a class of patients who have had magnificent sets of teeth up to the time of loss of health in middle life or old age. Then these almost perfect teeth commence rapidly to decay, and, with all our boasted skill, it is all but impossible to arrest the trouble.

In our younger patients a return to health after fevers often shows its beneficial result in the teeth. Impaired vitality at any time, from whatever cause, has its marked effect upon the dentine; and in this condition it becomes more thoroughly a culture medium, in which the caries fungi thrive. On the other hand, exposed dentine, from wear or from fracture, will often end in decay when the patient is in vigorous health.

Most of us remember the experiments of Dr. Robert Arthur, of Baltimore, on cut and polished dentine; I quote several of his observations from his published work entitled "Prevention of Decay of Teeth," published in Philadelphia by Lippincott in 1871: "The dentine has a power to protect itself. When the surface of the dentine becomes exposed by the loss of enamel, it grows darker in color and may be taken for decay, but so far from being affected in this way, it is really no longer liable to decay except under very unfavorable circumstances. A tooth that has undergone such a change usually remains free from decay during life."

"The dentine is thus shown to resist vitally the influence of the decomposing forces. There is a vital property in dentine by means of which it is endowed with a certain power of self-protection,—the contents of the *exposed* canals seem to have become changed into a hard ivory-like substance, and it is probable that in the contents of the canals there is an interchange of lime for organic tissue, and the new formation differs from normal dentine by becoming denser and discolored."

Dr. Horace Hayden, in his practice in Baltimore from 1804 to 1843, frequently filed out cavities in the front teeth of young children, and Dr. Arthur assures us that after a lapse of sixty-four years the filed surfaces of some of these teeth, which came under his observation, were entirely free from any trace of decay.

Dr. W. H. H. Thackston is on record as saying, 1871, "I frequently meet with pearly, soft teeth, that I filed freely twenty to twenty-five years ago, sound and good, with texture hardened by time, and the change that occurs in all dental and osseous tissue as we advance in life with surfaces shining and lustrous, as if some natural polishing process had been going on in the mouth during the interval."

Dr. Black's experiments show that progressive hardening of the dentine does not occur. In closing, I wish to call your attention to some of his conclusions, printed in the *Dental Cosmos* for May, 1895, page 415, and here let me say that in many of these conclusions I heartily agree with him. Let us consider this one, the fifth: "There is no basis for the supposition that the teeth of children under the age of twelve years are too soft to receive metallic fillings." In a sense I can agree with this; yet I believe it will have a very strong tendency to mislead. It is not merely a question of the softness of the tissue, but should be one rather of the health and vigor of the child and of our power as dental physicians to change the conditions which induce caries. Dr. Black has shown that the teeth of the young are by his tests the strongest, but in the opinion of nearly every operator whose judgment is of value it would in most cases be considered poor practice to insert gold fillings, however perfectly, at such an age. In my own practice I have for many years been a strong advocate of treatment of preparatory fillings made with the various cements. And twenty-five years' experience has shown me the solid value of this course.

It would be reasonable to expect failures in a majority of cases where teeth were filled with gold alone, and for the first time in the mouths of children under the twelfth year, even were it packed in the most perfect manner and by the finest operators.

I am wholly in accord with the seventh conclusion, where, in speaking of the hardness and strength of the teeth, he considers these qualities as dependent on the condition of the organic matrix. But I am at a loss to understand that part of the eighth conclusion where he says that differences in the strength (stated above as dependent upon the organic matrix) has no influence as to their liability to caries. This organic matrix, constituting the strength of the tooth, must, it seems to me, have a very strong influence as to its liability to decay, and I think this must be the conclusion at which we have all arrived. The organic matrix of the tooth is given a greater power to resist decay by constitutional factors during vigorous health. That it does not always succeed is not a proof of the absence of this vital force. This vital power is greatly lessened during almost all constitutional disturbances.

I am in accord with the statement, "That the cause of the difference in the liability of individuals to caries of the teeth is something in the constitution operating through the oral fluids and acting upon the active cause of caries, hindering or intensifying." But I would say more, I would have added, something in the constitution *operating through the organic structure of the dentine*, and also through the oral fluids, etc.

Decay, I believe, is in a measure dependent upon the condition of the organic structure of the tooth as well as on the condition of their environment. In proportion to the vitality of the part is its power to resist the action of external decomposing agents.

